# The predictive value of nontraditional lipid parameters for intracranial and extracranial atherosclerotic stenosis: a hospital-based observational study in China

**DOI:** 10.1186/s12944-022-01761-4

**Published:** 2023-01-28

**Authors:** Shun Yu, Lihong Yan, Junwei Yan, Xiaozhi Sun, Meixia Fan, Huanhuan Liu, Yongxin Li, Mingjin Guo

**Affiliations:** 1 Department of Vascular Surgery, Affiliated Hospital of Qingdao University, Qingdao, Shandong China; 2 Office of Hospital Director, Affiliated Hospital of Qingdao University, Qingdao, Shandong China

**Keywords:** Atherogenic index of plasma, Intracranial atherosclerotic stenosis, Remnant cholesterol, Lipoprotein combine index, Atherosclerosis, Ischemic stroke, Extracranial atherosclerotic stenosis

## Abstract

**Background:**

Ischemic strokes are primarily caused by intracranial and extracranial atherosclerotic stenosis. Nontraditional lipid parameters broaden traditional lipid profiles, better reflect the metabolism and interaction between different lipid components, and optimize the predictive ability of lipid profiles for atherosclerotic diseases. This research was carried out to investigate the predictive value of nontraditional lipid parameters for intracranial or extracranial atherosclerotic stenosis.

**Methods:**

The investigation collected data from inpatients who underwent cervical vascular ultrasonography, carotid CTA, cerebral artery CTA or MRA, and brain MRI or CT from December 2014 to December 2021. The nontraditional lipid parameters were calculated by collecting traditional lipid parameters. To evaluate the predictive power of nontraditional lipid parameters, logistic regression and receiver operating characteristic curve (ROC) analyses were performed.

**Results:**

Based on the inclusion and exclusion criteria, 545 patients were included. According to the imaging results, inpatients were divided into two groups, including no intracranial or extracranial atherosclerotic stenosis (*n* = 250) and intracranial or extracranial atherosclerotic stenosis (AS, *n* = 295). Among them, AS was further divided into three subgroups: intracranial atherosclerotic stenosis (ICAS), extracranial atherosclerotic stenosis (ECAS) and combined intracranial and extracranial atherosclerotic stenosis (IECAS). Logistic regression analysis showed that nontraditional lipid parameters, including the atherogenic index of plasma (AIP), remnant cholesterol (RC), nonhigh-density lipoprotein cholesterol (non-HDL-C), lipoprotein combine index (LCI), atherogenic coefficient (AC), Castelli’s index-I (CRI-I) and Castelli’s index-II (CRI-II), were significantly correlated with intracranial or extracranial atherosclerotic stenosis (*P* < 0.05). Compared with other nontraditional lipid parameters, regardless of adjusting for potential confounding factors, AIP had a greater OR value in ICAS (OR = 4.226, 95% CI: 1.681–10.625), ECAS (OR = 2.993, 95% CI: 1.119–8.003) and IECAS (OR = 4.502, 95% CI: 1.613–12.561). ROC curve analysis revealed that nontraditional lipid parameters had good predictive power for intracranial or extracranial atherosclerotic stenosis.

**Conclusions:**

This Chinese hospital-based study demonstrates that nontraditional lipid parameters (AIP, LCI, RC, CRI-II, AC, CRI-I and non-HDL-C) are effective predictors of intracranial and extracranial atherosclerotic stenosis, of which AIP may be a significant risk factor for predicting atherosclerotic arterial stenosis in the intracranial or extracranial regions.

**Supplementary Information:**

The online version contains supplementary material available at 10.1186/s12944-022-01761-4.

## Introduction

Stroke is the second leading cause of death and the third leading cause of death and disability combined worldwide, with ischemic strokes representing over half of all stroke types [1]. Ischemic strokes are primarily caused by intracranial and extracranial atherosclerotic stenosis [2–4]. It is paramount that extracranial and intracranial atherosclerotic stenosis be identified as early as possible to prevent ischemic strokes. Atherosclerotic disease risk can be predicted by traditional serum lipid parameters, which are widely used because of their easy collection process and low cost. Previous studies have demonstrated that traditional lipid parameters such as low-density lipoprotein cholesterol (LDL-C), total cholesterol (TC), triglycerides (TG), and high-density lipoprotein cholesterol (HDL-C) were considered to be correlated with cardiovascular disease or ischemic strokes [2, 5–11]. Among them, LDL-C, HDL-C, and TC were found to be linked to intracranial or extracranial carotid arterial stenosis [12–14].

Unlike traditional individual lipid parameters, nontraditional lipid parameters, such as the atherogenic index of plasma (AIP), TG/HDL-C, remnant cholesterol (RC), nonhigh-density lipoprotein cholesterol (non-HDL-C), lipoprotein combine index (LCI), atherogenic coefficient (AC), Castelli’s index-I (CRI-I) and Castelli’s index-II (CRI-II), are calculated from two or more traditional lipid parameters. Nontraditional lipid parameters provide insight into the balance between atherogenic lipoproteins and antiatherogenic lipoproteins, and several studies have shown that nontraditional lipid parameters such as AIP, non-HDL-C, LCI, RC, TG/HDL-C, AC, CRI-I and CRI-II were closely related to cardiovascular and cerebrovascular diseases, which might be even superior to traditional lipid parameters [6, 15–19].

The AIP is the base 10 logarithm of circulating TG/HDL-C, which is a plasma atherosclerosis marker proposed by Dobiás̆ová in 2001 [20]. The AIP value can be a straightforward means to indirectly reflect the level of sdLDL-C. SdLDL-C was defined as LDL-C particles with a diameter of less than 25.5 nm and a density of more than 1.034 g/mL [21], which was strongly atherogenic [22]. SdLDL-C has been listed as a potential biomarker of cardiovascular disease [23]. Studies have found that AIP is a novel marker of cardiovascular disease [24, 25] and may be superior to traditional lipid parameters [26–28]. Nevertheless, only a few studies have been conducted regarding the association between AIP and intracranial or extracranial atherosclerotic stenosis; the predictive value remains unclear for intracranial atherosclerotic stenosis [29, 30].

Remnant cholesterol refers to the cholesterol content in a subset of triglyceride-rich lipoproteins known as remnants, viz. chylomicron remnants, VLDL, and IDL during nonfasting conditions, and VLDL and IDL during fasting conditions [31]. LCI was also a new nontraditional lipid parameter that was calculated as the ratio of TC∗TG∗LDL-C to HDL-C. Previous investigations on LCI and RC have mainly focused on the field of cardiovascular disease [32, 33], and studies of RC and LCI with extracranial and intracranial atherosclerotic stenosis are very rare.

The relationships between various nontraditional lipid parameters and intracranial or extracranial atherosclerotic arterial stenosis are still unclear. Logistic regression and ROC curves were utilized to analyze the relationships between various nontraditional lipid parameters and intracranial or extracranial atherosclerotic stenosis. A total of 545 inpatients were involved in this research. For more accurate assessment of atherosclerotic stenosis in the intracranial or extracranial regions, multiple imaging examinations were performed.

## Methods

### Study population

This is observational clinical research conducted at a single center with a total of 545 inpatients involved (Additional file 1: Fig. S1). Patients were divided into two groups, including no intracranial or extracranial artery stenosis (the control group, *n* = 250) and intracranial or extracranial atherosclerotic stenosis (the study group, *n* = 295). The study group consisted of three subgroups: intracranial atherosclerotic stenosis (ICAS, *n* = 108), combined intracranial and extracranial artery stenosis (IECAS, *n* = 113), and extracranial artery stenosis (ECAS, *n* = 74). Furthermore, the extracranial atherosclerotic stenosis group consisted of extracranial carotid atherosclerotic stenosis (ECCAS, *n* = 45), extracranial vertebral atherosclerotic stenosis (*n* = 40), and combined extracranial carotid and vertebral atherosclerotic stenosis (*n* = 25). Data for the study were provided by the Medical Research Big Data Platform of the Affiliated Hospital of Qingdao University, with full approval from the Ethics Committee.

### Definition

All included patients underwent carotid artery imaging (carotid CTA and cervical vascular ultrasonography), cerebral artery imaging (cerebral artery CTA or MRA), and brain CT or MRI.

Intracranial and extracranial atherosclerotic stenosis could be defined as follows:

1. Intracranial atherosclerotic stenosis: significant atherosclerotic stenosis of cerebral arteries in the proximal anterior, middle, or posterior areas; the internal carotid, vertebral, or basilar arteries in the intracranial region exhibited obvious atherosclerotic stenosis.
2. Extracranial atherosclerotic stenosis: common carotid artery, extracranial region of internal carotid artery, or extracranial segment of vertebral artery had obvious stenosis
3. Combined intracranial and extracranial atherosclerotic stenosis: significant stenosis was present in both the intracranial and extracranial arteries in tandem or parallel lesions;
4. No intracranial or extracranial arterial stenosis: no obvious stenosis of either intracranial or extracranial arteries;
5. Significant artery stenosis of intracranial or extracranial regions could be defined as ≥50% stenosis of arteries in the intracranial or extracranial regions [34, 35] or an internal carotid artery flow velocity ≥ 125 cm/s [36].

For patients with different imaging results between cerebral artery CTA and MRA, CTA was given priority due to the limitations of MRA imaging. All imaging findings were assessed by three experienced doctors who were blinded to this study.

### Data

Following admission, blood samples from the peripheral veins were collected early in the morning for biochemical testing after 8 hours of fasting. In addition, clinical baseline data of inpatients were collected, such as age, sex, body mass index (BMI), history of hypertension, smoking, ischemic stroke, diabetes mellitus and coronary heart disease.

An automatic biochemical analyzer was utilized to measure LDL-C, TC, TG, and fasting blood glucose (FBG) levels. The following formulas were used to calculate nontraditional lipid parameters:

1. AIP = lg (TG/HDL − C) [20];
2. non − HDL − C = TC − HDL − C [16];
3. AC = non − HDL − C/HDL − C [30];
4. CRI − I = TC/HDL − C [30];
5. CRI − II = LDL − C/HDL − C [30];
6. LCI = TC ∗ TG ∗ LDL − C/HDL − C [32];
7. RC = TC − HDL − C − LDL − C [37].

### Statistical analyses

Normally distributed continuous variables are presented as the mean ± standard deviation, which was compared between two groups by using an independent samples *t* test. In cases where continuous variables were not normally distributed, the variables are shown as medians and interquartile ranges (IQRs), and the Mann–Whitney U test was applied for comparison. Categorical variables are presented as frequencies (percentages). Analyses of categorical variables were conducted using *chi*-square tests. To assess the relationship between the nontraditional lipid parameters and intracranial or extracranial atherosclerotic stenosis, logistic regression analyses were utilized. Furthermore, three regression models were constructed: Model 1 (without adjusting for any covariates), Model 2 (adjusted for age and sex) and Model 3 (Model 2 plus BMI, FBG, history of coronary heart disease, hypertension, smoking, ischemic stroke, and diabetes mellitus); in the models, the nontraditional lipid parameters were separated into four quartiles to assess the relationship between quartiles and intracranial or extracranial artery stenosis with the lowest quartile as a reference. ROC curves were applied to calculate the area under the curve (AUC) of nontraditional lipid parameters for each group. All data analysis was performed using IBM SPSS Statistics 26.0. *P* < 0.05 was considered statistically significant.

## Results

### Univariate analysis

#### Comparison of study and control groups

As shown in Table 1, data for clinical baseline characteristics and lipid parameters of subjects are summarized. Compared to the control group, the study group had significant differences in history of hypertension, ischemic stroke, smoking and diabetes mellitus (*P* < 0.05). HDL-C was also found to be lower in the study group (*P* < 0.05). Age, FBG and other lipid parameters were found to be markedly increased in the study group (*P* < 0.05).

**Table 1:** Clinical baseline characteristics and lipid parameters of subjects

Variable	Control (*n* = 250)	Study group (*n* = 295)	*P*	ICAS (*n* = 108)	IECAS (*n* = 113)	ECAS (*n* = 74)
Age	63.65 ± 10.01	67.96 ± 9.87^a^	< 0.001	66.50(57.00–72.00)	70.00(63.50–76.00) ^a^	69.00(64.00–76.00) ^a^
Gender(male)	175(70.0%)	83(71.9%)	0.633	62(57.4%)^a^	90(79.6%)	60(81.1%)
Smoking	84(33.6%)	147(49.8%)^a^	< 0.001	41(38.0%)	64(56.6%)^a^	42(56.8%)^a^
Hypertension	117(46.8%)	191(64.7%)^a^	< 0.001	69(63.9%)^a^	80(70.8%)^a^	42(56.8%)
Diabetes mellitus	52(20.8%)	92(31.2%)^a^	0.006	27(25.0%)	42(37.2%)^a^	23(31.1%)
Coronary heart disease	46(18.4%)	49(16.6%)	0.583	21(19.4%)	17(15.0%)	11(14.9%)
Ischemic stroke	68(27.2%)	127(43.1%)^a^	< 0.001	50(46.3%)	53(46.9%)^a^	24(32.4%)
BMI	24.95(23.10–27.03)	25.70(22.90–27.80)	0.054	25.95(24.05–28.33) ^a^	25.70(22.70–27.65)	24.50 (22.00–27.42)
LDL-C	2.37 (1.88–2.93)	3.00 (2.57–3.52) ^a^	< 0.001	2.99 (2.57–3.61) ^a^	3.08 (2.73–3.51) ^a^	2.84 (2.28–3.55) ^a^
TC	4.23 (3.59–4.96)	4.80 (4.25–5.51) ^a^	< 0.001	4.83 (4.19–5.65) ^a^	4.87 (4.39–5.53) ^a^	4.72 (4.13–5.34) ^a^
TG	1.14 (0.84–1.66)	1.51 (1.14–2.02) ^a^	< 0.001	1.61 (1.05–2.24) ^a^	1.48 (1.27–1.96) ^a^	1.46 (1.10–1.87) ^a^
HDL-C	1.25 (1.04–1.46)	1.17 (1.00–1.36) ^a^	0.001	1.17 (1.00–1.35) ^a^	1.13 (0.99–1.35) ^a^	1.19 (0.99–1.38)
FBG	5.12 (4.66–5.98)	5.38(4.78–7.02) ^a^	0.001	5.36 (4.77–7.03) ^a^	5.43 (4.85–7.96) ^a^	5.19 (4.56–6.69)
AIP	−0.04(− 0.23–0.16)	0.12 (− 0.03–0.27) ^a^	< 0.001	0.12 (− 0.05–0.30) ^a^	0.12 (− 0.01–0.26) ^a^	0.11 (−0.05–0.26) ^a^
non-HDL-C	2.86 (2.34–3.58)	3.64 (3.14–4.25) ^a^	< 0.001	3.64 (3.13–4.33) ^a^	3.69 (3.23–4.36) ^a^	3.51 (2.92–4.10) ^a^
AC	2.30 (1.76–3.06)	3.15 (2.63–3.79) ^a^	< 0.001	3.05 (2.57–3.68) ^a^	3.20 (2.71–3.88) ^a^	3.16 (2.38–3.60) ^a^
CRI-I	3.30 (2.76–4.06)	4.15 (3.63–4.79) ^a^	< 0.001	4.05 (3.57–4.68) ^a^	4.20 (3.71–4.88) ^a^	4.16 (3.38–4.60) ^a^
CRI-II	1.88 (1.47–2.55)	2.57 (2.18–3.15) ^a^	< 0.001	2.56 (2.18–3.14) ^a^	2.63 (2.26–3.25) ^a^	2.58 (1.92–3.05) ^a^
LCI	8.99 (4.95–17.85)	20.06(12.26–30.49) ^a^	< 0.001	20.49(11.84–31.28) ^a^	20.85(14.10–31.91) ^a^	17.00(11.37–26.03) ^a^
RC	0.49 (0.35–0.71)	0.60 (0.47–0.77) ^a^	< 0.001	0.60 (0.45–0.79) ^a^	0.61 (0.47–0.79) ^a^	0.58 (0.48–0.73) ^a^

“^a^”means compared with the control group, *P* < 0.05. *ICAS* intracranial atherosclerotic stenosis, *ECAS* extracranial atherosclerotic stenosis, *IECAS* combined intracranial and extracranial atherosclerotic stenosis, *BMI* body mass index, *FBG* fasting blood glucose, *LDL-C* low-density lipoprotein cholesterol, *TC* total cholesterol, *TG* triglyceride, *HDL-C* high-density lipoprotein cholesterol, *AIP* atherogenic index of plasma, *non-HDL-C* nonhigh-density lipoprotein cholesterol, *AC* atherogenic coefficient, *CRI-I* Castelli’s index-I, *CRI-II* Castelli’s index-II, *LCI* lipoprotein combine index, *RC* remnant cholesterol

#### Subgroup analysis

In comparison to the control group, significant differences were found in sex, history of hypertension and BMI in the ICAS group. On the other hand, age, history of smoking, hypertension, diabetes mellitus and ischemic stroke had significant differences in the IECAS (*P* < 0.05). Traditional lipid parameters (LDL-C, TC and TG) and nontraditional lipid parameters (including non-HDL-C, AIP, AC, RC, CRI-I and CRI-II) and FBG were found to be significantly increased in ICAS and IECAS; in contrast, HDL-C levels were lower in ICAS and IECAS (*P* < 0.05). Marked differences were shown in age and the history of smoking between the ECAS and the control group, and LDL-C, TC, TG, AIP, AC, LCI, CRI-I, CRI-II, non-HDL-C and RC were markedly increased in ECAS (*P* < 0.05). In accordance with the subgroup analysis of the extracranial artery stenosis group (ECCAS), significant differences could be seen in age and the history of smoking in ECCAS compared to the control group. Levels of LDL-C and TC were markedly increased in ECCAS (*P* < 0.05), and non-HDL-C, LCI, AC, CRI-I and CRI-II values were markedly greater in ECCAS (*P* < 0.05) (Table 1 and Additional file 2: Table S1).

### Logistic regression analyses

Three logistic regression models were constructed, and the results are shown in Additional file 3: Table S2 - Additional file 6: Table S5. Regardless of the adjustment of confounding factors, an independent relationship was observed between nontraditional lipid parameters and atherosclerotic arterial stenosis in the intracranial or extracranial regions, and AIP had the largest OR value compared with other lipid parameters.

**Fig. 1:**
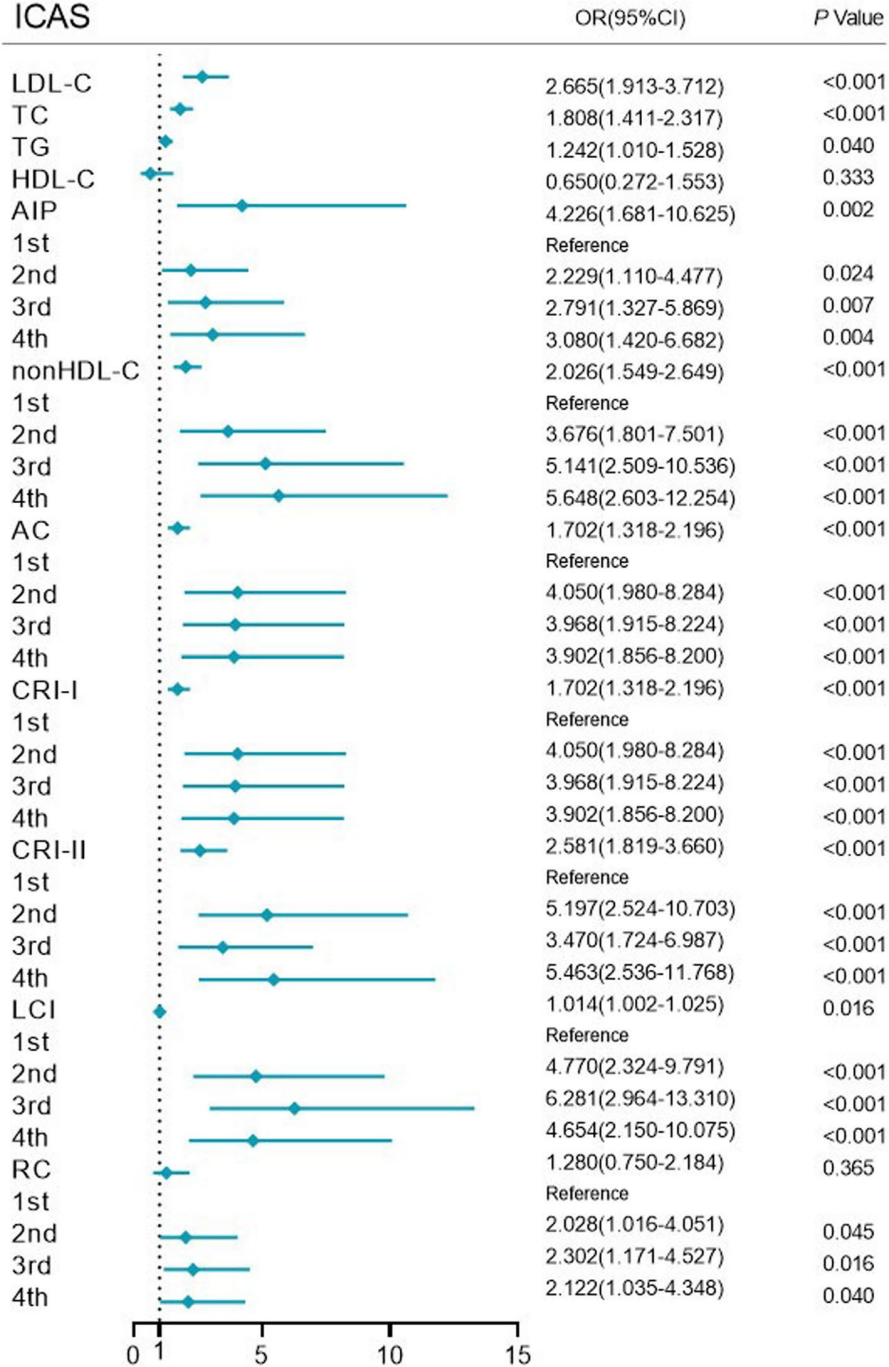
Forest plot of logistic regression analysis of intracranial atherosclerotic stenosis in Model 3. Data were adjusted for age, sex, BMI, FBG, history of ischemic stroke, coronary heart disease, smoking, hypertension, and diabetes mellitus. ICAS intracranial atherosclerotic stenosis, BMI body mass index, FBG fasting blood glucose, LDL-C low-density lipoprotein cholesterol, TC total cholesterol, TG triglyceride, HDL-C high-density lipoprotein cholesterol, AIP atherogenic index of plasma, non-HDL-C nonhigh-density lipoprotein cholesterol, AC atherogenic coefficient, CRI-I Castelli’s index-I, CRI-II Castelli’s index-II, LCI lipoprotein combine index, RC remnant cholesterol

**Fig. 2:**
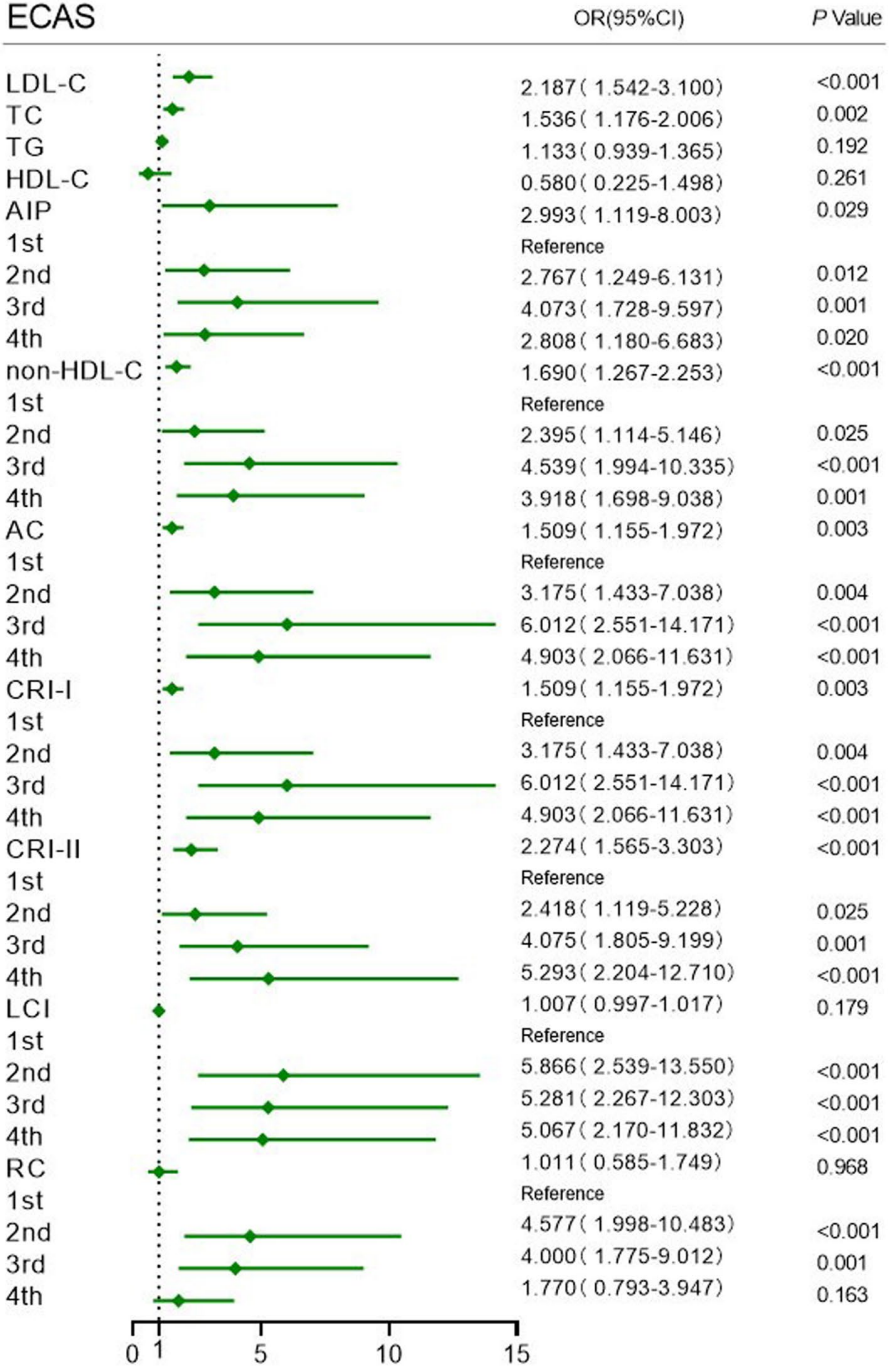
Forest plot of logistic regression analysis of extracranial atherosclerotic stenosis in Model 3. Data were adjusted for age, sex, BMI, FBG, history of ischemic stroke, coronary heart disease, smoking, hypertension, and diabetes mellitus. ECAS extracranial atherosclerotic stenosis, BMI body mass index, FBG fasting blood glucose, LDL-C low-density lipoprotein cholesterol, TC total cholesterol, TG triglyceride, HDL-C high-density lipoprotein cholesterol, AIP atherogenic index of plasma, non-HDL-C nonhigh-density lipoprotein cholesterol, AC atherogenic coefficient, CRI-I Castelli’s index-I, CRI-II Castelli’s index-II, LCI lipoprotein combine index, RC remnant cholesterol

In model 3, the ICAS group indicated the following: LDL-C (OR = 2.665, 95% CI: 1.913–3.712), TC (1.808, 1.411–2.317), TG (1.242, 1.010–1.528), and nontraditional lipid parameters, including AIP (4.226, 1.681–10.625), non-HDL-C (2.026, 1.549–2.649), AC (1.702, 1.318–2.196), CRI-I (1.702, 1.318–2.196), CRI-II (2.581, 1.819–3.660), and LCI (1.014, 1.002–1.025), were significantly correlated with intracranial atherosclerotic stenosis (all *P* < 0.05), and AIP had the highest OR value. Analysis of the ECAS group showed that LDL-C (OR = 2.187, 95% CI: 1.542–3.100), TC (1.536, 1.176–2.006) and nontraditional lipid parameters, including AIP (2.993, 1.119–8.003), non-HDL-C (1.690, 1.267–2.253), AC (1.509, 1.155–1.972), CRI-I (1.509, 1.155–1.972), and CRI-II (2.274, 1.565–3.303), were all significantly associated with extracranial atherosclerotic stenosis (all *P* < 0.05). With the lowest quartile as a reference, the 2^nd^, 3^rd^ and 4^th^ quartiles of LCI were highly correlated with extracranial artery stenosis (*P* < 0.05), and the 2^nd^ and 3^rd^ quartiles of RC were statistically significantly correlated with extracranial artery stenosis (*P* < 0.05) (Figs. 1 and 2).

### ROC curve analysis

ROC curves revealed that AIP, non-HDL-C, LCI, AC, RC, CRI-I and CRI-II had greater AUC values in IECAS, ICAS and ECAS, while the AUC values of non-HDL-C, AC, CRI-I, CRI-II and LCI were greater in ECCAS (Table 2).

**Table 2:** AUC in ROC curve for nontraditional lipid parameters

Group/variable	AUC	Standard error	95% confidence interval
**ICAS**			
**AIP**	0.663	0.030	0.605–0.722
**non-HDL-C**	0.723	0.027	0.670–0.776
**AC**	0.734	0.026	0.682–0.785
**CRI-I**	0.734	0.026	0.682–0.785
**CRI-II**	0.739	0.027	0.687–0.791
**LCI**	0.737	0.027	0.684–0.790
**RC**	0.612	0.031	0.551–0.674
**ECAS**			
**AIP**	0.628	0.036	0.557–0.699
**non-HDL-C**	0.668	0.034	0.602–0.735
**AC**	0.684	0.034	0.617–0.751
**CRI-I**	0.684	0.034	0.617–0.751
**CRI-II**	0.689	0.035	0.621–0.758
**LCI**	0.685	0.033	0.620–0.750
**RC**	0.599	0.034	0.533–0.666
**IECAS**			
**AIP**	0.680	0.028	0.625–0.736
**non-HDL-C**	0.725	0.027	0.671–0.779
**AC**	0.744	0.027	0.691–0.796
**CRI-I**	0.744	0.027	0.691–0.796
**CRI-II**	0.754	0.027	0.702–0.806
**LCI**	0.744	0.026	0.692–0.795
**RC**	0.618	0.031	0.557–0.679
**ECCAS**			
**AIP**	0.582	0.045	0.494–0.670
**non-HDL-C**	0.680	0.040	0.602–0.758
**AC**	0.654	0.043	0.570–0.738
**CRI-I**	0.654	0.043	0.570–0.738
**CRI-II**	0.665	0.044	0.577–0.752
**LCI**	0.669	0.040	0.590–0.748
**RC**	0.582	0.039	0.506–0.657

*ICAS* intracranial atherosclerotic stenosis, *ECAS* extracranial atherosclerotic stenosis, *IECAS* combined intracranial and extracranial atherosclerotic stenosis, *ECCAS* extracranial carotid atherosclerotic stenosis, *BMI* body mass index, *FBG* fasting blood glucose, *LDL-C* low-density lipoprotein cholesterol, *TC* total cholesterol, *TG* triglyceride, *HDL-C* high-density lipoprotein cholesterol, *AIP* atherogenic index of plasma, *non-HDL-C* nonhigh-density lipoprotein cholesterol, *AC* atherogenic coefficient, *CRI-I* Castelli’s index-I, *CRI-II* Castelli’s index-II, *LCI* lipoprotein combine index, *RC* remnant cholesterol. *ROC* receiver operating characteristic, *AUC* area under the curve

## Discussion

Through the above study, these findings demonstrated that (1) the AIP may represent an independent risk factor for intracranial or extracranial atherosclerotic stenosis; (2) the LCI and RC were closely related to intracranial or extracranial atherosclerotic stenosis; and (3) the AC, CRI-I and CRI-II were highly correlated with intracranial or extracranial artery stenosis, whereas the AC, CRI-I and CRI-II were more effective at predicting ICAS or IECAS.

This study found that regardless of the subgroup, AIP was significantly related to intracranial and extracranial artery stenosis. AIP is a novel nontraditional lipid parameter with values ranging from negative to positive, and values of 0 are closely associated with LDL particles with a diameter of 25.5 nm; that size corresponds to the cutoff value of particle size between the two phenotypes of LDL particles A and B [20]. Since sdLDL-C is predominant in phenotype B [38], AIP can be used as an indicator of the LDL phenotype and indirectly reflect sdLDL-C levels. The low affinity of sdLDL-C to its receptor leads to a longer blood circulation time, which increases the possibility of its atherosclerotic modification in plasma; this longer circulation time, coupled with its small particle size, makes it easier to invade the arterial wall, thus participating in atherogenesis [22, 39–42]. Compared with LDL-C levels, sdLDL-C levels might serve as a better indicator of coronary heart disease [39, 43]. At the same time, sdLDL-C was also closely associated with ischemic stroke, carotid atherosclerosis and carotid stenosis [22, 44–48]. Unfortunately, sdLDL-C is limited in clinical practice as a result of the high detection cost and complicated procedures, but AIP can only be used as a simple method to reflect the level of sdLDL-C, and many studies have confirmed that AIP can predict the occurrence of cardiovascular disease events [49–51]. Nevertheless, a very small number of studies have investigated the relationships between AIP and intracranial or extracranial atherosclerosis [29, 30].

Analyses conducted on ICAS showed that AIP might represent an independent predictor of intracranial artery stenosis in this study. Interestingly, a previous study found that there was an association between AIP and the prevalence of asymptomatic intracranial artery stenosis, but the statistical significance disappeared when potential confounders were fully adjusted [30]. Although this finding seems to be slightly different from the previous study, perhaps it can be explained by the following reasons: (1) the two investigations are based on different study populations; (2) the prevalence of ICAS was higher in this study; (3) this study included all asymptomatic and symptomatic patients with arterial stenosis, and coronary heart disease as a covariate that might have influenced the study results was adjusted; and (4) transcranial Doppler ultrasonography (TCD) was applied to evaluate intracranial artery stenosis in the former and was excessively dependent on the operator, so the accuracy and reliability of its examination might be limited. By using CTA or MRA and cervical vascular ultrasonography, intracranial or extracranial artery stenosis was fully evaluated in this study. In addition, this study indicated that AIP was independently correlated with extracranial carotid atherosclerotic stenosis. Recently, case–controlled research carried out at a single center using a sample of patients who suffered from ischemic stroke found that with quartile 1 as the reference, the 3^rd^ and 4^th^ quartiles of AIP were independently correlated with symptomatic carotid artery stenosis [29]. With the lowest quartile as a reference in this study, the 3^rd^ quartile of AIP was closely associated with extracranial carotid atherosclerotic stenosis regardless of adjustment for potential confounding variables. In contrast to the former study, the research included all symptomatic and asymptomatic patients excluded from lipid-lowering therapy and adjusted for confounding variables in Models 2 and 3, particularly adjusting for multiple potential confounding variables in Model 3. Thus, this research concluded that AIP may be an independent predictor of extracranial carotid atherosclerotic stenosis.

Both LCI and RC were closely associated with intracranial and extracranial atherosclerotic stenosis. A case–control study showed that compared with patients without coronary artery disease, patients who underwent bypass grafting for cardiovascular disease had higher levels of LCI, and an ROC curve analysis showed that its specificity was the highest [33]. Si *et al.* found that increased retinol-binding protein-4 (RBP-4) and LCI values were independent risk factors for acute coronary syndrome (ACS), and the combination of the two tests might be a potential diagnostic indicator for ACS [32]. It is worth noting that the analysis of LCI demonstrated that even though the OR value of LCI in logistic regression analysis of each group was small, close to the cutoff value of 1, LCI had a large OR in this study during logistic regression analysis of LCI quartiles, and ROC curve analysis showed good predictive values. Therefore, the LCI may be used as an independent predictor of intracranial or extracranial atherosclerotic arterial stenosis. The estimated value of RC can be simply calculated by subtracting LDL-C and HDL-C from TC [37]. Previous studies have reported that even after reducing LDL-C to recommended levels, a larger residual cardiovascular disease risk remained, which might be partly due to increased residual cholesterol levels [31, 37], and the causal relationship between RC and coronary heart disease has been established in multiple studies [31, 52, 53]. A Korean case–control study demonstrated that high levels of remnant lipoprotein cholesterol (RLP-C) were correlated with ischemic stroke, particularly atherosclerotic stroke of the large arteries [54]. Subsequently, a prospective multicenter study in Korea revealed that reduced RLP-C was correlated with preventing the angiographic progression of symptomatic ICAS [55]. Yang *et al.* found that RC/HDL-C was significantly associated with intracranial atherosclerotic stenosis [56]. Therefore, according to the above studies, this investigation hypothesized that RC was significantly associated with ICAS. Fortunately, especially in the extracranial carotid stenosis subgroup, the results confirmed the speculation that RC also showed a significant difference in the quartile analysis of each group, indicating that RC may be a risk factor independently associated with atherosclerotic stenosis, whether intracranial or extracranial.

Lipid ratios, including AC, CRI-I and CRI-II, were shown to be closely correlated with intracranial or extracranial atherosclerotic arterial stenosis in this investigation. Interestingly, based on our findings, CRI-I and AC have the same OR values, confidence intervals and *P* values. In essence, both CRI-I and AC have the same statistical significance, as the calculation formulas of the two can be converted into each other. Thus, the author recommends that only one of the two is applied in scientific research or clinical practice. It is noteworthy that CRI-II had a larger OR value than CRI-I or AC in each group, suggesting that CRI-II was more strongly associated with intracranial and extracranial atherosclerotic stenosis. This may be because Castelli’s index-II better reflects the balance between LDL-C, which is the major atherogenic lipoprotein, and HDL-C, which is the antiatherogenic lipoprotein. As has been reported previously, CRI-II, AC or CRI-I have been shown to be closely correlated with ischemic stroke [57], carotid intima-media thickness [58–61], carotid plaque [61–63] and intracranial atherosclerotic stenosis [30, 56]. Yang and colleagues reported that CRI-II, AC and CRI-I were closely correlated with ICAS but not with ECAS after adjusting for confounders [56]. In a subsequent community-based study of asymptomatic intracranial artery stenosis, a significant association was also reported between CRI-II AC and CRI-I and an increased prevalence of asymptomatic intracranial artery stenosis [30]. However, this investigation demonstrated that CRI-II, AC and CRI-I had a strong relationship with intracranial or extracranial atherosclerotic stenosis, whether or not adjusted for confounders. This finding seems to contradict the conclusion of Yang and colleagues, and the following reasons may explain why: (1) there is a higher prevalence of extracranial artery stenosis in this study, and the investigation excluded patients taking lipid-lowering treatment, which may well influence the findings; (2) this study included subjects with total occlusion of the intracranial and extracranial arteries, considering subjects with complete intracranial and extracranial arterial occlusion who may have more severe dyslipidemia. This investigation revealed that CRI-II, AC and CRI-I were independent predictors of intracranial or extracranial atherosclerotic stenosis. At the same time, it seemed that CRI-II, AC and CRI-I were more valuable for predicting ICAS or IECAS.

There are several novelties in this study. First, LCI and RC were rarely studied in intracranial and extracranial atherosclerotic stenosis, and the research found that LCI and RC were significantly correlated with intracranial and extracranial atherosclerotic stenosis through both univariate and multivariate analyses. Second, it was also examined whether nontraditional lipid parameters (AIP, LCI, RC, CRI-II, AC, CRI-I and non-HDL-C) could predict IECAS, and the findings showed that nontraditional lipid parameters were closely related to IECAS. Therefore, this research draws broader conclusions that nontraditional lipid parameters (AIP, LCI, RC, CRI-II, AC, CRI-I, and non-HDL-C) are independent risk factors for atherosclerotic stenosis in the intracranial or extracranial regions. Furthermore, since previous studies have not established the predictive value of AIP for intracranial and extracranial atherosclerotic arterial stenosis, the research demonstrated that AIP was an independent predictor of atherosclerotic arterial stenosis in the intracranial or extracranial regions.

### Study strengths and limitations

#### Strengths

(1) This study collected detailed imaging data and integrated several imaging results, so it evaluated intracranial and external arterial stenosis more accurately. (2) Compared with vascular ultrasonography, nontraditional lipid parameters are less operator-dependent and easy to collect; thus, they can also be utilized as a screening method for atherosclerotic arterial stenosis in the intracranial or extracranial regions. (3) Subjects taking lipid-lowering drugs were excluded from this study, and the logistic regression model was adjusted to include history of coronary heart disease and other confounding factors to minimize the interference of these confounding factors on the study results.

#### Limitations

(1) This is a case–control investigation conducted by a single center based on hospital patients, and the causal relationship is limited. However, it is based on hospital patients for whom we have complete and accurate imaging examinations of intracranial and extracranial arteries, which can fully evaluate intracranial and extracranial artery stenosis. (2) Our study is based on the Chinese population, and it has been reported that AIP is related to race, region, diet and lifestyle, so caution should be taken in extending our study conclusions.

(3) Previous studies reported that AIP was related to serum uric acid. Unfortunately, we did not collect serum uric acid data from patients, so we did not analyze it.

## Conclusion

This investigation demonstrated that AIP, LCI, RC, CRI-II, AC, CRI-I and non-HDL-C were markedly correlated with intracranial and extracranial atherosclerotic stenosis. The AIP may be a better independent predictor of atherosclerotic arterial stenosis in the intracranial or extracranial regions. Nontraditional lipid parameters may be used as an alternative method to imaging examination in the early screening of intracranial and extracranial atherosclerotic stenosis.

### Supplementary Information

**Additional file 1: Fig. S1.** Flow chart of study.

**Additional file 2: Table S1.** Clinical baseline characteristics and lipid parameters of four study groups.

**Additional file 3: Table S2.** Logistic regression analysis of intracranial atherosclerotic stenosis.

**Additional file 4: Table S3.** Logistic regression analysis of extracranial atherosclerotic stenosis.

**Additional file 5: Table S4.** Logistic regression analysis of combined intracranial and extracranial atherosclerotic stenosis.

** Additional file 6: Table S5.** Logistic regression analysis of extracranial carotid atherosclerotic stenosis.

## Data Availability

The datasets used and/or analyzed during the current study are available from the first author on reasonable request.

## Abbreviations

AIP: Atherogenic index of plasma
AC: Atherogenic coefficient
CRI-I: Castelli’s index-I
CRI-II: Castelli’s index-II
Non-HDL-C: Nonhigh-density lipoprotein cholesterol
LCI: Lipoprotein combine index
RC: Remnant cholesterol
AUC: Area under the curve
LDL-C: Low-density lipoprotein cholesterol
TC: Total cholesterol
TG: Triglyceride
HDL-C: High-density lipoprotein cholesterol
sdLDL-C: Small dense low-density lipoprotein cholesterol
AS: Intracranial or extracranial atherosclerotic stenosis
CTA: Computed tomography angiography
CT: Computed Tomography
MRA: Magnetic resonance angiography
MRI: Magnetic resonance imaging
ROC: Receiver operating characteristic
ICAS: Intracranial atherosclerotic stenosis
ECAS: Extracranial atherosclerotic stenosis
IECAS: Combined intracranial and extracranial atherosclerotic stenosis
ECCAS: Extracranial carotid atherosclerotic stenosis
BMI: Body mass index
FBG: Fasting blood glucose
IQR: Interquartile range
OR: Odds ratio
95% CI: 95% confidence interval
