# Supplementary figures and images for: The predictive value of nontraditional lipid parameters for intracranial and extracranial atherosclerotic stenosis: a hospital-based observational study in China
Source: Lipids Health Dis. 2023 Jan 28;22:16. doi: 10.1186/s12944-022-01761-4 (PMC9883878; doi:10.1186/s12944-022-01761-4)

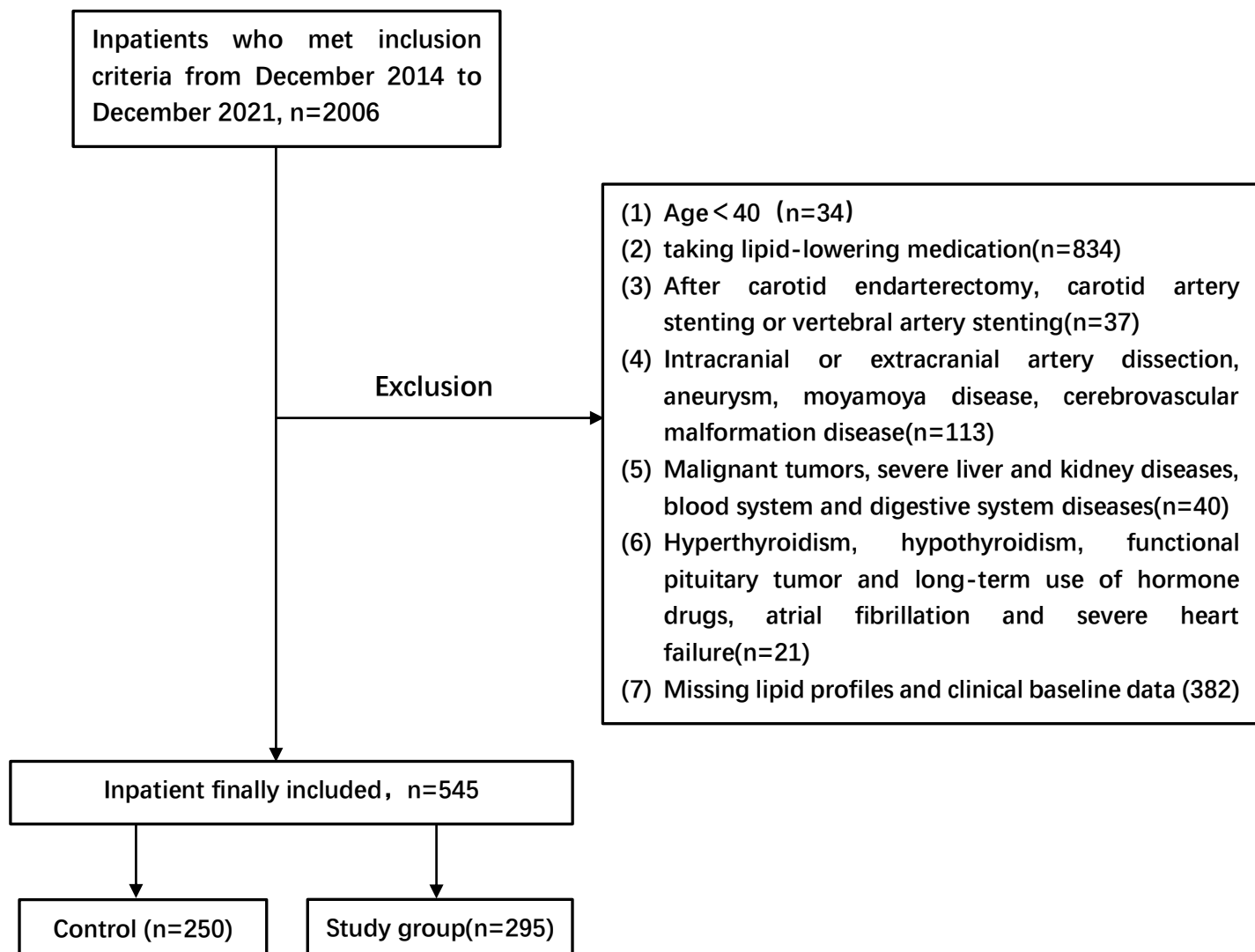

Supplement: Supplementary file 1 — Additional file 1: Fig. S1. Flow chart of study. [file 12944_2022_1761_MOESM1_ESM.pdf]
