# Supplementary material for: The predictive value of nontraditional lipid parameters for intracranial and extracranial atherosclerotic stenosis: a hospital-based observational study in China
Source: Lipids Health Dis. 2023 Jan 28;22:16. doi: 10.1186/s12944-022-01761-4 (PMC9883878; doi:10.1186/s12944-022-01761-4)
Supplement: Supplementary file 2 — Additional file 2: Table S1. Clinical baseline characteristics and lipid parameters of four study groups. [file 12944_2022_1761_MOESM2_ESM.docx]

| Table S1 Clinical baseline characteristics and lipid parameters of four study groups | | | | | |
| --- | --- | --- | --- | --- | --- |
| Variable | **ICAS**  **（*n =* 108）** | **IECAS**  **（*n* = 113）** | **ECAS**  **（*n* = 74）** | **ECCAS**  **（*n* = 45）** | ****P*** |
| Age | 66.50(57.00-72.00) | 70.00(63.50-76.00) ***** | 69.00(64.00-76.00) ***** | 69.20±10.17***** | 0.026***** |
| Gender（male） | 62(57.4%) ***** | 90(79.6%) | 60(81.1%) | 34(75.6%) | <0.001***** |
| Smoking | 41(38.0%) | 64(56.6%) ***** | 42(56.8%) ***** | 23(51.1%) ***** | 0.022***** |
| Hypertension | 69(63.9%) ***** | 80(70.8%) ***** | 42(56.8%) | 26(57.8%) | 0.196 |
| Diabetes mellitus | 27(25.0%) | 42(37.2%) ***** | 23(31.1%) | 9(20.0%) | 0.100 |
| Coronary heart disease | 21(19.4%) | 17(15.0%) | 11(14.9%) | 8(17.8%) | 0.796 |
| Ischemic stroke | 50(46.3%) | 53(46.9%) ***** | 24(32.4%) | 14(31.1%) | 0.075 |
| BMI | 25.95(24.05-28.33) ***** | 25.70(22.70-27.65) | 24.50(22.00-27.42) | 25.20(22.25-27.05) | 0.027***** |
| LDL-C | 2.99 (2.57-3.61) ***** | 3.08 (2.73-3.51) ***** | 2.84 (2.28-3.55) ***** | 2.87 (2.29-3.58) ***** | 0.318 |
| TC | 4.83 (4.19-5.65) ***** | 4.87 (4.39-5.53) ***** | 4.72 (4.13-5.34) ***** | 4.78 (4.30-5.39) ***** | 0.619 |
| TG | 1.61 (1.05-2.24) ***** | 1.48 (1.27-1.96) ***** | 1.46 (1.10-1.87) ***** | 1.46 (1.03-1.84) | 0.284 |
| HDL-C | 1.17 (1.00-1.35) ***** | 1.13 (0.99-1.35) ***** | 1.19 (0.99-1.38) | 1.27 (1.11-1.43) | 0.196 |
| FBG | 5.36 (4.77-7.03) ***** | 5.43 (4.85-7.96) ***** | 5.19 (4.56-6.69) | 5.01 (4.40-6.53) | 0.010***** |
| AIP | 0.12 (-0.05-0.30) ***** | 0.12 (-0.01-0.26) ***** | 0.11 (-0.05-0.26) ***** | 0.08(-0.12-0.21) | 0.326 |
| non-HDL-C | 3.64 (3.13-4.33) ***** | 3.69 (3.23-4.36) ***** | 3.51 (2.92-4.10) ***** | 3.53 (2.82-4.16) ***** | 0.338 |
| AC | 3.05 (2.57-3.68) ***** | 3.20 (2.71-3.88) ***** | 3.16 (2.38-3.60) ***** | 2.90 (2.24-3.50) ***** | 0.151 |
| CRI-I | 4.05 (3.57-4.68) ***** | 4.20 (3.71-4.88) ***** | 4.16 (3.38-4.60) ***** | 3.90 (3.24-4.50) ***** | 0.151 |
| CRI-II | 2.56 (2.18-3.14) ***** | 2.63 (2.26-3.25) ***** | 2.58 (1.92-3.05) ***** | 2.49 (1.77-3.04) ***** | 0.249 |
| LCI | 20.49(11.84-31.28) ***** | 20.85(14.10-31.91) ***** | 17.00(11.37-26.03) ***** | 16.00(10.46-27.34) ***** | 0.091 |
| RC | 0.60 (0.45-0.79) ***** | 0.61 (0.47-0.79) ***** | 0.58 (0.48-0.73) ***** | 0.57 (0.47-0.69) | 0.615 |
| “*”means compared with the control group，*P*<0.05. **P* indicates comparison among four study groups, *P*<0.05. ICAS intracranial atherosclerotic stenosis, ECAS extracranial atherosclerotic stenosis, IECAS combined intracranial and extracranial atherosclerotic stenosis, ECCAS extracranial carotid atherosclerotic stenosis, BMI body mass index, FBG fasting blood glucose, LDL-C low-density lipoprotein cholesterol, TC total cholesterol, TG triglyceride, HDL-C high-density lipoprotein cholesterol, AIP atherogenic index of plasma, non-HDL-C nonhigh-density lipoprotein cholesterol, AC atherogenic coefficient, CRI-I Castelli's index-I, CRI-II Castelli's index-II, LCI lipoprotein combine index, RC remnant cholesterol. | | | | | |
