# Supplementary material for: The predictive value of nontraditional lipid parameters for intracranial and extracranial atherosclerotic stenosis: a hospital-based observational study in China
Source: Lipids Health Dis. 2023 Jan 28;22:16. doi: 10.1186/s12944-022-01761-4 (PMC9883878; doi:10.1186/s12944-022-01761-4)
Supplement: Supplementary file 3 — Additional file 3: Table S2. Logistic regression analysis of intracranial atherosclerotic stenosis. [file 12944_2022_1761_MOESM3_ESM.docx]

| Table S2 Logistic regression analysis of intracranial atherosclerotic stenosis | | | | | | |
| --- | --- | --- | --- | --- | --- | --- |
| ICAS | **Model 1** |  | **Model 2** |  | **Model 3** |  |
| Variable | **OR（95%CI）** | ***P*** | **OR（95%CI）** | ***P*** | **OR（95%CI）** | ***P*** |
| LDL-C | 2.436(1.813- 3.274) | <0.001 | 2.444(1.811- 3.297) | <0.001 | 2.665(1.913-3.712) | <0.001 |
| TC | 1.646(1.334-2.032) | <0.001 | 1.627(1.313-2.017) | <0.001 | 1.808(1.411-2.317) | <0.001 |
| TG | 1.346(1.117-1.621) | 0.002 | 1.398(1.148-1.701) | 0.001 | 1.242(1.010-1.528) | 0.040 |
| HDL-C | 0.419(0.200-0.880) | 0.022 | 0.282(0.126-0.629) | 0.002 | 0.650(0.272-1.553) | 0.333 |
| AIP | 5.735(2.635-12.485) | <0.001 | 7.921(3.484-18.011) | <0.001 | 4.226(1.681-10.625) | 0.002 |
| 1st | **Reference** | | | | | |
| 2nd | 2.321(1.245-4.328) | 0.008 | 2.811(1.473-5.366) | 0.002 | 2.229(1.110-4.477) | 0.024 |
| 3rd | 3.154(1.657-6.003) | <0.001 | 3.771(1.929-7.371) | <0.001 | 2.791(1.327-5.869) | 0.007 |
| 4th | 3.514(1.830-6.747) | <0.001 | 4.953(2.466-9.9470 | <0.001 | 3.080(1.420-6.682) | 0.004 |
| non-HDL-C | 1.963(1.545-2.494) | <0.001 | 1.965(1.541-2.505) | <0.001 | 2.026(1.549-2.649) | <0.001 |
| 1st | **Reference** | | | | | |
| 2nd | 3.294(1.747- 6.211) | <0.001 | 3.406(1.791-6.474) | <0.001 | 3.676(1.801-7.501) | <0.001 |
| 3rd | 4.257(2.244- 8.076) | <0.001 | 4.571(2.382-8.768) | <0.001 | 5.141(2.509-10.536) | <0.001 |
| 4th | 5.765(2.903- 11.449) | <0.001 | 5.398(2.672-10.907) | <0.001 | 5.648(2.603-12.254) | <0.001 |
| AC | 1.913(1.512-2.420) | <0.001 | 2.033(1.594-2.593) | <0.001 | 1.702(1.318-2.196) | <0.001 |
| 1st | **Reference** | | | | | |
| 2nd | 4.081(2.145-7.765) | <0.001 | 4.426(2.288-8.564) | <0.001 | 4.050(1.980-8.284) | <0.001 |
| 3rd | 4.441(2.318-8.510) | <0.001 | 5.375(2.732-10.574) | <0.001 | 3.968(1.915-8.224) | <0.001 |
| 4th | 5.393(2.763-10.525) | <0.001 | 6.148(3.072-12.305) | <0.001 | 3.902(1.856-8.200) | <0.001 |
| CRI-I | 1.913(1.512-2.420) | <0.001 | 2.033(1.594-2.593) | <0.001 | 1.702(1.318-2.196) | <0.001 |
| 1st | **Reference** | | | | | |
| 2nd | 4.081(2.145-7.765) | <0.001 | 4.426(2.288-8.564) | <0.001 | 4.050(1.980-8.284) | <0.001 |
| 3rd | 4.441(2.318-8.510) | <0.001 | 5.375(2.732-10.574) | <0.001 | 3.968(1.915-8.224) | <0.001 |
| 4th | 5.393(2.763-10.525) | <0.001 | 6.148(3.072-12.305) | <0.001 | 3.902(1.856-8.200) | <0.001 |
| CRI-II | 2.929(2.117-4.053) | <0.001 | 3.164(2.263-4.422) | <0.001 | 2.581(1.819-3.660) | <0.001 |
| 1st | **Reference** | | | | | |
| 2nd | 5.333(2.754-10.327) | <0.001 | 5.556(2.830-10.907) | <0.001 | 5.197(2.524-10.703) | <0.001 |
| 3rd | 4.000(2.117-7.556) | <0.001 | 4.501(2.342-8.649) | <0.001 | 3.470(1.724-6.987) | <0.001 |
| 4th | 8.000(3.943-16.232) | <0.001 | 8.688(4.207-17.945) | <0.001 | 5.463(2.536-11.768) | <0.001 |
| LCI | 1.019(1.008-1.030) | <0.001 | 1.020(1.009-1.031) | <0.001 | 1.014(1.002-1.025) | 0.016 |
| 1st | **Reference** | | | | | |
| 2nd | 3.756(1.991-7.088) | <0.001 | 4.887(2.500-9.553) | <0.001 | 4.770(2.324-9.791) | <0.001 |
| 3rd | 5.500(2.819-10.731) | <0.001 | 6.440(3.214-12.905) | <0.001 | 6.281(2.964-13.310) | <0.001 |
| 4th | 5.704(2.912-11.170) | <0.001 | 6.496(3.224-13.088) | <0.001 | 4.654(2.150-10.075) | <0.001 |
| RC | 1.558(0.957-2.535) | 0.074 | 1.596(0.954-2.670) | 0.075 | 1.280(0.750-2.184) | 0.365 |
| 1st | **Reference** | | | | | |
| 2nd | 1.966(1.048-3.689) | 0.035 | 2.135(1.125-4.049) | 0.020 | 2.028(1.016-4.051) | 0.045 |
| 3rd | 2.283(1.229-4.241) | 0.009 | 2.517(1.338-4.734) | 0.004 | 2.302(1.171-4.527) | 0.016 |
| 4th | 2.296(1.203-4.382) | 0.012 | 2.451(1.269-4.732) | 0.008 | 2.122(1.035-4.348) | 0.040 |
| Model 1: Unadjusted；Model 2: Adjusted for age and gender；Model 3：Adjusted for age, gender, BMI, FBG, the history of smoking, hypertension, diabetes mellitus, coronary heart disease, and ischemic stroke. ICAS intracranial atherosclerotic stenosis, BMI body mass index, FBG fasting blood glucose, LDL-C low-density lipoprotein cholesterol, TC total cholesterol, TG triglyceride, HDL-C high-density lipoprotein cholesterol, AIP atherogenic index of plasma, non-HDL-C non-high-density lipoprotein cholesterol, AC atherogenic coefficient, CRI-I Castelli's index-I, CRI-II Castelli's index-II, LCI lipoprotein combine index, RC remnant cholesterol. | | | | | | |
